# EP-YOLO: A Printed Circuit Board Defect Detection Network Integrating Coordinate Attention and Multi-Level Gradient Flow Optimization

**DOI:** 10.3390/s26103106

**Published:** 2026-05-14

**Authors:** Xiangsuo Fan, Can Yang, Ling Yu

**Affiliations:** 1 School of Automation, Guangxi University of Science and Technology, Liuzhou 545006, China; 100002085@gxust.edu.cn (X.F.); 20240203014@stdmail.gxust.edu.cn (C.Y.); 2 Earthwork Collaborative Innovation Center, Liuzhou 545006, China; 3 Engineering Research Center of Advanced Engineering Equipment, University of Guangxi, Liuzhou 545006, China; 4 Guangxi Low-Altitude Unmanned Aircraft Key Technologies Engineering Research Center, Liuzhou 545616, China

**Keywords:** defect detection, PCB, small goals, YOLOv8, deep learning

## Abstract

The dependence of electronic products on printed circuit boards (PCBs) is increasing. In recent years, PCB defect detection technology has achieved considerable results, but the defect targets are small, the background is complex, and there are many integrated components, which poses great challenges to product quality control. Therefore, this paper proposes a printed circuit board defect detection network that integrates coordinate attention and multi-level gradient flow optimization, called EP-YOLO, to improve the detection accuracy of printed circuit board defects. Firstly, this paper improves the model’s ability to capture small target details by reconstructing the detection head of the small target. Based on this, a Shallow Context Feature Extraction (SCFE) network is designed to fuse shallow features with multi-scale features, effectively preventing the loss of shallow detail information and texture features. At the same time, in order to suppress background noise, this paper designs a multi-level feature gradient flow optimization module (C2f_CA, abbreviated as CCA) that integrates coordinate attention and a Cross-Stage Partial Frequency-domain Omni-Kernel module (CSPFOK) to enhance feature extraction capability. Finally, the SCYLLA-IoU (SIoU) optimization model training process was introduced. The experimental results showed that EP-YOLO achieved 97.5% mAP50 on the PKU-Market-PCB dataset and 98.5% mAP50 on the DeepPCB dataset, with a parameter reduction of approximately 12.55%, outperforming popular object detection networks. The results highlight the potential capabilities of EP-YOLO, providing a powerful and effective solution for industrial PCB defect detection.

## 1. Introduction

As an essential part of electronic devices, the production process of PCBs is very complex and prone to small defects such as mouse bites, open circuits, and short circuits [1]. PCB surface defects are characterized by diverse types, low feature resolution, and easy blending with the background, which poses a serious challenge to defect detection. Especially for minor defects, they often only occupy a very small area of the image (often less than 0.16% of the total pixels), and even slight defects can easily be overlooked during the detection process; however, accurate detection of these minor defects is crucial for ensuring the reliability of electronic devices. Traditional manual visual inspection is inefficient and susceptible to interference, while detection based on electrical or infrared methods is costly and difficult to achieve batch automated detection [2]. It can be seen that in PCB quality control, how to develop an efficient and accurate automated visual inspection solution has always been a focus of attention in the industry.

Early object detection techniques mostly relied on non-deep image-processing algorithms and classical machine learning strategies [3]. These methods typically include techniques such as threshold segmentation [4], edge detection [5], morphological processing, and template matching [6], as well as detection methods based on feature extraction using classifiers [7]. For example, in specific controllable environments, these methods can locate certain types of defects, but often struggle to achieve high robustness. Traditional methods mainly rely on image-processing techniques, usually using a sliding window framework combined with underlying visual operators for implementation. This type of method starts from basic feature point matching or region similarity calculation, and directly completes the detection task through pixel level operations. However, relying on traditional detection methods often requires a large amount of computation and is time-consuming, making it difficult to balance real-time detection with accuracy in industrial environments. Therefore, there is an urgent need to adopt more advanced technologies to replace these methods.

With the development of deep learning, object detection methods have rapidly advanced and can automatically extract salient features [8]. These methods mainly consist of single-stage and two-stage algorithms [9]. Among them, two-stage algorithms (such as R-CNN [10], Fast R-CNN [11], Faster R-CNN [12]) first generate candidate regions for the image, and then classify and regress each candidate box. These methods have high accuracy but high computational complexity and slow speed. Relatively speaking, single-stage algorithms such as YOLO series [13] and SSD [14] directly perform end-to-end prediction on the original image, with a simple detection process and fast speed, and are therefore widely used in industrial scenarios. It should be noted that although single-stage methods have obvious speed advantages, their performance in small-object detection is usually not as good as two-stage algorithms. Therefore, in practical applications, a compromise between speed and accuracy is often needed. Recently, Wang et al. [15] have improved the detection efficiency and accuracy by improving the feature extraction mode and loss function. Wu et al. [16] used window attention-based cross-scale convolution fusion to enhance multi-scale defect feature representation.

Although these methods have made progress, PCB defect features exhibit extremely high concealment and complexity [17]. In addition, the uncertainty of the imaging environment further weakens the stability of detection [18]. These methods perform poorly in suppressing background noise and struggle to maintain robustness in complex industrial scenarios [19].

This paper comprehensively optimizes the feature extraction, feature fusion, and detection modules of YOLOv8 to address the challenges faced in PCB defect detection. The main contributions are as follows:

1. This paper proposes an improved network based on YOLOv8, named EP-YOLO. This network not only improves detection accuracy but also reduces parameter size, demonstrating strong innovation.

2. In response to the shortcomings of feature extraction and fusion in YOLOv8, this paper first designs the SCFE module. The SCFE module performs spatially sensitive downsampling on the shallow features (P2/P3) of YOLOv8, preserves detailed information, and enhances feature transfer to the neck, effectively improving the boundary localization accuracy and spatial integrity detection capability of small defects. Subsequently, the CSPFOK module was proposed, which organically integrates multi-scale directional convolution, frequency-domain feature enhancement, and cross-stage local attention mechanism, improving the texture perception ability of the model for small defects and the modeling efficiency of multi-directional spatial context information. The designed CCA module effectively enhances the sensitivity of feature maps to positional information and the ability of information exchange between channels.

3. In order to enhance the detection capability for small targets, the detection head of YOLOv8 has been redesigned with the addition of a P2 layer. To reduce the loss of semantic and positional information caused by frequent downsampling in large-object detection heads, this paper removes the P5 layer detection head, thereby improving the accuracy of small-object defect recognition and reducing redundant calculations.

4. In order to more accurately characterize the positional relationship between targets and improve the accuracy of boundary box regression, this study uses Scylla-IoU (SIoU) loss as the regression loss function. By jointly optimizing angle deviation and distance shape constraints, the convergence speed and robustness of boundary localization for small defects are significantly improved.

## 2. Related Work

### 2.1. Industrial Defect Detection Method Based on Traditional Image Processing and Machine Learning

Traditional image-processing algorithms and machine learning methods have made significant contributions to industrial defect detection. Firstly, traditional manually designed feature extraction algorithms, such as the Directional Gradient Histogram (HOG) proposed by Dalal et al. [20], improve the accuracy of pedestrian detection by calculating the distribution of local gradient orientations, and combine it with linear support vector machines to form a classic framework. For surface defect detection, Ojala et al. [21] combined the local binary pattern algorithm with texture feature encoding and achieved significant results. The improved version of the algorithm has been widely used in the field of industrial quality inspection. The centrosymmetric LBP (CS-LBP) operator proposed by Heikkilä et al. [22] achieves a breakthrough in processing speed in metal surface defect detection by simplifying the feature dimension. These methods generally adopt the technical route of collaborative optimization between feature engineering and machine learning, such as Lowe’s team [23] introducing a feature pyramid matching strategy on the SIFT algorithm, which significantly improves the performance of scale invariant detection. However, such methods require manually designed and complex feature extraction rules and have limited adaptability to complex scenes such as target deformation and occlusion.

In order to overcome the limitations of traditional image-processing algorithms, researchers have proposed many machine learning-based algorithms that have been applied to PCB defect detection research. For example, Ko and Cho [24] combined a learning vector quantization (LVQ) neural network [25] and a fuzzy logic [26] scheme to detect solder joints, reducing computational complexity while increasing the clarity of complex boundaries. Belbachir et al. [27] proposed a PCB defect detection system using wavelet transform (WVT) and multilayer perceptron (MLP) neural network. The system extracts features from images captured by a CCD camera and inputs them into the corresponding trained neural network. Wu and Zhang et al. [28] used color gradients and Boolean rules to detect solder joint defects. By deducing the relationship between solder joint types and solder joint areas, Boolean rules are established to classify solder joints. Although machine learning-based methods have achieved good performance, detection performance largely depends on the quality of previously extracted features. Meanwhile, each method is designed for specific applications and has poor generalization ability and robustness.

### 2.2. Industrial Defect Detection Method Based on Deep Learning

One of the main challenges in PCB defect detection is the large number and extremely small size of defect targets [29]. Due to frequent downsampling in CNN networks, small defects may almost disappear in deep features, making detectors prone to missed detections. In recent years, researchers have proposed various specially designed detection networks to address this issue. For example, Ding et al. [30] proposed TDD Net, which is specifically designed for detecting small defects in PCBs and has achieved high detection accuracy. However, the model is relatively large and difficult to deploy. In terms of single-stage methods, Hou et al. [31] proposed the EffNet PCB detection framework, which optimizes the accuracy and real-time performance of PCB defect detection through advanced feature fusion. Liu et al. [32] designed Robot-YOLOv5 for robot scenarios, incorporating the C3Ghost module into the original YOLOv5 framework to significantly reduce model parameters and computational complexity, thereby achieving faster inference speed and more convenient deployment in robot environments. Xiao et al. [33] proposed a lightweight FCA module that effectively enhances the spatial dependency modeling and long-range feature interaction capabilities of industrial surface defect images by aggregating average and salient features in both vertical and horizontal directions. In recent years, YOLO optimization methods for small PCB defects have emerged one after another. The AD-YOLO proposed by Zhou et al. [34] combines the Swin Transformer module with an efficient channel space attention mechanism (ECSA) into YOLOv7, and adds a small target detection layer (SODL) to enhance the feature extraction and localization capabilities for small targets in complex airport scenes. The YOLO-HMC network designed by Yuan et al. [35] integrates the HorNet module, MCBAM channel attention, and the CARAFE upsampling mechanism, significantly improving its detection accuracy in a compact layout. The above studies have improved the detection performance of small defects by improving the network structure and feature enhancement strategies. End-to-end detection models such as DETR [36] and RT-DETR [37] further simplify the detection process, avoiding post-processing steps such as Non Maximum Suppression (NMS) by directly outputting defect location and category. They have great potential in high-precision PCB detection scenarios.

Some researchers have also optimized the network structure. For example, Tan et al. [38] from Google Research proposed a weighted bidirectional feature pyramid network (BiFPN) to enhance the detection capability of multi-scale targets. Another series of works utilizes lightweight variants, such as MobileNet modules, to replace standard convolutions and construct lightweight deep neural networks, in order to reduce model size and improve inference speed [39]. Overall, single-stage algorithms such as YOLOv5, YOLOv8, and their improvements have been widely used in industrial PCB inspection, with detection speeds typically reaching tens to hundreds of FPS, while gradually approaching or even surpassing two-stage methods in accuracy through architectural improvements. The above methods still show limitations in small-object defect detection and model generalization, and there is still room for improvement.

## 3. Materials and Methods

### 3.1. Overall Framework Diagram

**Figure 1:**
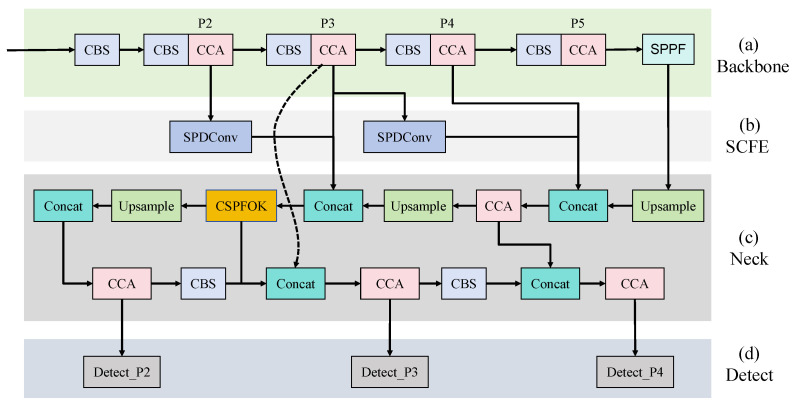
Detailed EP-YOLO network architecture diagram: (**a**) backbone network; (**b**) SCFE network; (**c**) feature fusion network; (**d**) detection head.

This study proposes an improved model EP-YOLO based on YOLOv8, which is mainly applied to industrial PCB defect detection. The overall structure of EP-YOLO is shown in Figure 1. Firstly, this study reconstructed the detection head by adding a detection head P2 with a resolution of 160 × 160. P2 increases the projected area of each small target on the feature map by four times, avoiding the compression of small targets into a very small number of pixels during downsampling, which could lead to information loss. At the same time, the 20 × 20 resolution large-object detection head (P5) in the original YOLOv8 model is removed to achieve model lightweighting and reduce redundant calculations. Then, the introduction of the CCA module and CSPFOK module enhances the representational ability of spatial and frequency-domain features of defects. Secondly, in the feature fusion stage, an SCFE network is designed to further improve the information exchange mechanism of multi-scale features by enhancing shallow semantic representation. Finally, SIoU is introduced to optimize the model training process.

### 3.2. CCA

**Figure 2:**
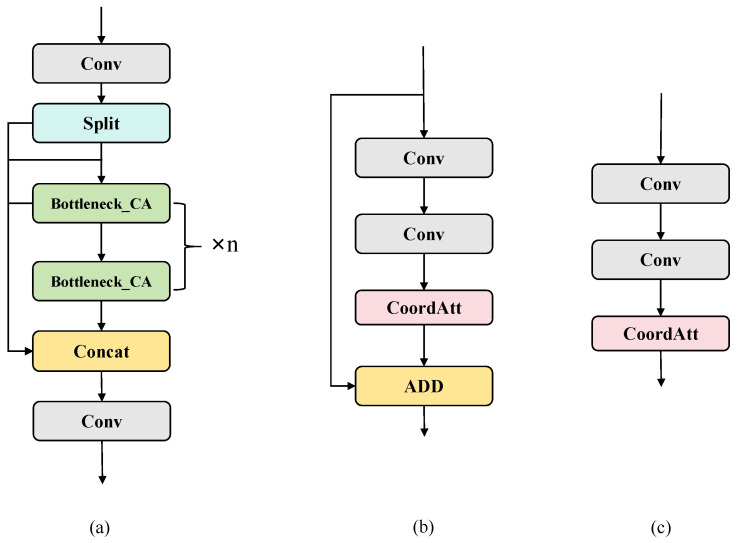
(**a**) The structure of CCA; (**b**) Bottleneck_CA (structure = True); (**c**) Bottleneck_CA (structure = False).

The backbone of YOLOv8 mainly consists of a stack of simple convolution modules. In PCB defect detection tasks, defects usually have characteristics such as small size, fine edges, and similarity to background texture. This leads to the local response of small defects being easily masked by background or large-scale structures, resulting in the gradual weakening of defect boundaries and location information. In order to address the above issues, this paper introduces CoordAtt attention [40] within C2f to design a new module (CCA), which targets the characteristics of PCB defects, such as a high proportion of small targets, sensitive spatial position information, and strong background interference. As shown in Figure 2.

**Figure 3:**
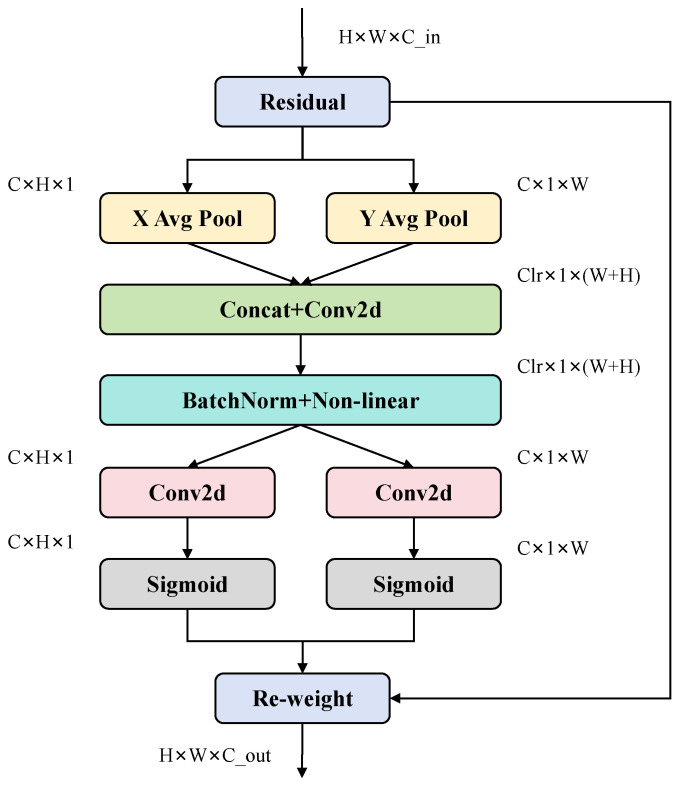
CoordAttention structure.

Traditional attention mechanisms only focus on modeling information in the channel dimension, ignoring the correlation between spatial structures. However, CoordAtt introduces a direction sensitive attention mechanism to jointly model channel features and spatial position information, effectively improving the network’s responsiveness and spatial perception accuracy to key regions while capturing horizontal and vertical global dependencies. Its structure is shown in Figure 3.

The CoordAtt attention mechanism can be understood as two collaborative sub-processes: the explicit position-encoding stage and the direction-guided attention construction stage. CoordAtt reconstructs the traditional two-dimensional global aggregation operation into a single axis-oriented serialized feature compression mechanism. By performing one-dimensional aggregation along both horizontal and vertical directions, it preserves spatial position information while enhancing directional sensitivity, achieving dynamic integration of low-dimensional embedding and spatial perception. This lays the dual foundation of structural prior and directional dependence for subsequent attention weight generation, thereby preserving fine spatial structures and improving long-range dependency modeling capabilities. The mathematical expression for global average pooling is as follows, where $z_{c}$ denotes the scalar output of the global spatial information corresponding to the *c*-th channel. $x_{c}\left(i,j\right)$ is the pixel value of the *c*-th channel in the input feature map with coordinates (i,j).

(1) $$z_{c}=\frac{1}{H\times W}\sum_{i=1}^{H}\sum_{j=1}^{W}x_{c}\left(i,j\right)$$

Specifically, assuming the input is $X\in R^{C\times H\times W}$, CoordAtt first applies global average pooling in both vertical and horizontal directions to form a biaxial structural perception path. $z_{c}^{h}\left(h\right)$ and $z_{c}^{w}\left(w\right)$ are the component outputs of the *c*-th channel at height and width, respectively.

(2) $$\begin{array}{c} z_{c}^{h}\left(h\right)=\frac{1}{W}\sum_{i=0}^{W-1}x_{c}\left(h,i\right) \end{array}$$

(3) $$\begin{array}{c} z_{c}^{w}\left(w\right)=\frac{1}{H}\sum_{j=0}^{H-1}x_{c}\left(j,w\right) \end{array}$$

These two one-dimensional feature paths encode spatial information in the vertical and horizontal directions and achieve directional separation of positional encoding through axial context extraction. Subsequently, they are concatenated into an intermediate feature representation with the shape of $X\in R^{C/r\times (H+W)}$ which is used for subsequent attention weight generation and spatial dependency modeling, where r is the channel compression ratio. Through a set of 1×1 convolution operations and a joint mapping with a nonlinear activation function, the two axial features are effectively fused to generate an intermediate feature representation with directional perception and semantic integration capabilities, providing a unified low-dimensional feature basis for subsequent attention generation.

(4) $$f=\delta \left(Conv_{1\times 1}\left(\left[z^{h},z^{w}\right]\right)\right)$$

Among them, [$z^{h},z^{w}$] represents concatenating vertical and horizontal vectors, and δ represents nonlinear activation function. Subsequently, the fused intermediate features are decoupled into two independent branches in the vertical and horizontal directions according to the original direction, and input into two 1×1 convolution operators to restore the channel dimension consistent with the original input. Among them, σ represents the Sigmoid activation function.

(5) $$g^{h}=\sigma \left(Conv_{1\times 1}\left(f^{h}\right)\right)$$

(6) $$g^{w}=\sigma \left(Conv_{1\times 1}\left(f^{w}\right)\right)$$

Finally, the generated vertical and horizontal attention maps $g^{h}$ and $g^{w}$ are applied to the input feature map element by element to achieve dual axis saliency guidance. The final output result can be represented as a feature map with direction enhancement. By combining the information from two attention paths, precise enhancement of key regions and effective suppression of irrelevant backgrounds can be achieved, thereby improving the discriminative and localization capabilities of feature representation.

(7) $$y_{c}\left(i,j\right)=x_{c}\left(i,j\right)\times g_{c}^{h}\left(i\right)\times g_{c}^{w}\left(j\right)$$

where $g_{c}^{h}\left(i\right)$ denotes the vertical attentional weight of the cth channel on the *i*-th row and $g_{c}^{w}\left(j\right)$ denotes the horizontal attentional weight of the cth channel on the jth column.

### 3.3. SCFE

**Figure 4:**
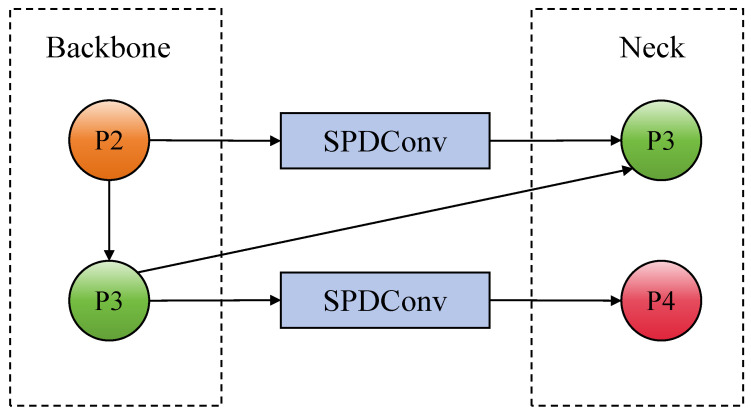
Network schematic diagram of SCFE.

In PCB defect detection tasks, defect targets usually have the characteristics of small size and sparse distribution. Many defects, such as excessive copper and burrs, often exhibit highly similar texture features to the background copper foil or circuit structure at the microscale, resulting in blurred semantic boundaries and low local contrast. This may lead to early loss of key information in the shallow feature extraction stage of traditional YOLOv8, thereby restricting the overall detection performance. To effectively address the above difficulties, this paper designs a Shallow Context Feature Extraction (SCFE) network, whose structure is shown in Figure 4.

**Figure 5:**
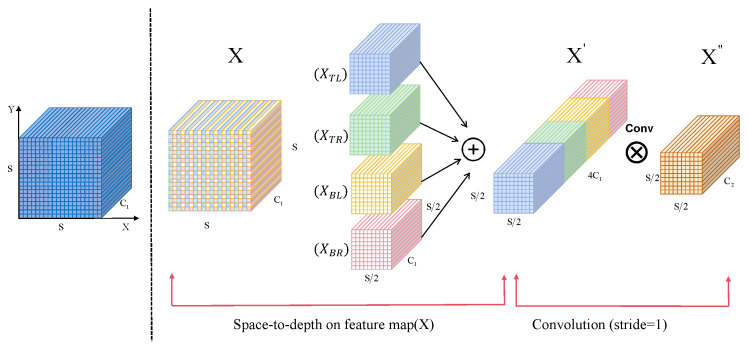
Detailed SPDConv structure.

The SCFE module performs target-oriented remodeling on the shallow feature maps P2 and P3. The SCFE module introduces two SPDConvs [41] to preserve spatially sensitive detail information for downsampling, effectively compressing low-level semantically significant but high-resolution feature maps and transmitting them to the neck feature fusion path. The detailed SPDConv structure is shown in Figure 5.

Let the input feature map be $X\in R^{S\times S\times C_{1}}$, where S denotes the spatial size and $C_{1}$ denotes the number of channels. The SPD layer segments *X* into four localized regions corresponding to the top-left, top-right, bottom-left, and bottom-right sections of the image. These regions are subsequently reorganized into four sub-feature tensors, each with dimensions of (S/2, S/2, $C_{1}$), formulated as follows:

(8) $$\begin{array}{c} X_{\mathrm{TL}}=X\left[:,0:\frac{s}{2},0:\frac{s}{2}\right] \end{array}$$

(9) $$\begin{array}{c} X_{\mathrm{TR}}=X\left[:,0:\frac{s}{2},\frac{\;s}{2}:s\right] \end{array}$$

(10) $$\begin{array}{c} X_{\mathrm{BL}}=X\left[:,\frac{s}{2}:s,0:\frac{s}{2}\right] \end{array}$$

(11) $$\begin{array}{c} X_{\mathrm{BR}}=X\left[:,\frac{s}{2}:s,\frac{\;s}{2}:s\right] \end{array}$$

The four feature maps are concatenated along the channel dimension to obtain a new feature map $X^{\prime }$, which is then processed by a standard non-strided convolution layer. Compress the channel dimension from the original $4C_{1}$ to $C_{2}$ ($C_{2}$ < $4C_{1}$), and outputs the final feature map $X^{\prime \prime }$.

(12) $$X^{\prime \prime }={\mathrm{Conv}}_{s=1}\left(X^{\prime }\right)$$

This convolutional layer does not change the spatial resolution, but uses learnable convolution kernels to filter and fuse the increased channel information, thereby highlighting local textures and edge details related to PCB defects. The SPDConv module ensures a reasonable reduction in the overall feature space scale during the downsampling process throughout the entire SCFE process, while fully mining and preserving fine-grained information in high-resolution features, enabling subsequent detection heads to more accurately distinguish subtle differences between defects and complex backgrounds.

### 3.4. CSPFOK

**Figure 6:**
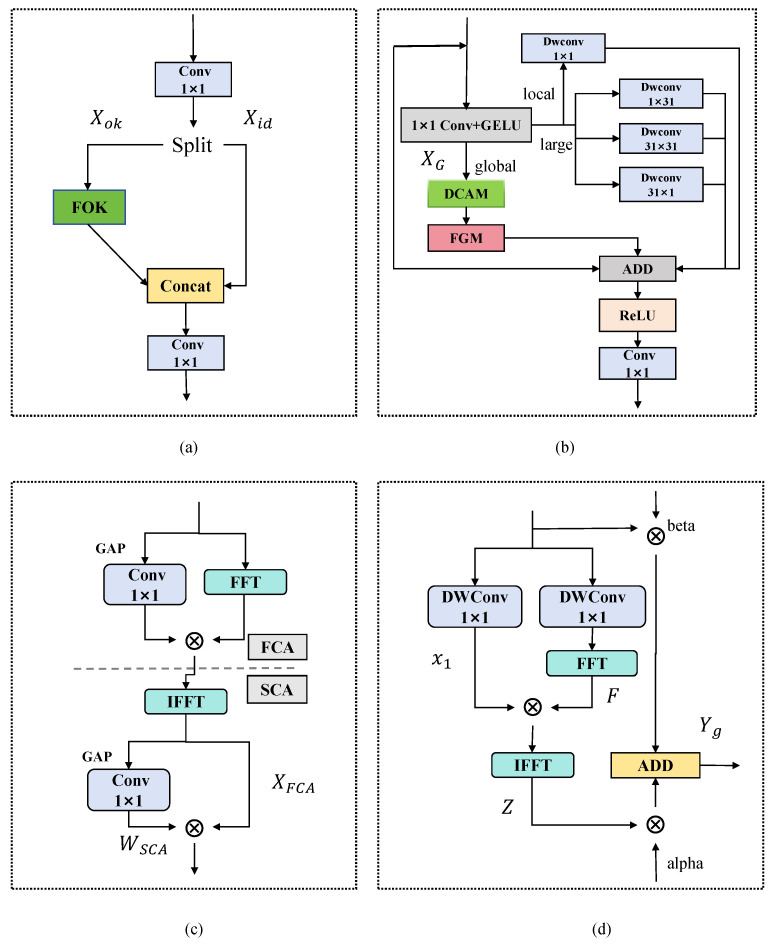
Detailed CSPFOK structure: (**a**) CSPFOK; (**b**) FOK; (**c**) DCAM; (**d**) FGM.

Due to the diverse sizes and irregular shapes of PCB defects, the fusion effect between shallow and deep features is limited when the model performs multi-scale feature fusion. Therefore, based on the Omni-Kernel module [42], this study proposes a Cross-Stage Partial Frequency-domain Omni-Kernel module (CSPFOK), as shown in Figure 6a. Realize more efficient feature representation with limited computational cost. The CSPFOK module integrates the CSP [43] concept with the multi-scale convolution and frequency-domain attention mechanism of FOK, achieving collaborative perception and joint modeling of local detail structures and global semantic context, and promoting deep fusion of features between spatial and semantic levels.

The CSPFOK module divides the input features into two subpaths: $X_{id}$ is directly retained as the information transfer path, and $X_{ok}$ enters into the subsequent backbone branch for the depth transformation. The two sub-tensors [eC,(1 − *e*)*C*] correspond to the FOK branch and the Identity branch, where e is a hyperparameter belonging to the range between (0, 1) (in this paper, we take e=0.25).

Figure 6b shows the schematic diagram of FOK. Given the input features $X_{ok}$, after 1 × 1 Conv, FOK divides the input features into three parallel subpaths. The features extracted from the three branches are subsequently fused by element-wise addition and passed through the ReLU activation function to further integrate the information and enhance the nonlinear representation of the features. Meanwhile, the combination of residual connection and attention mechanism helps to alleviate the gradient vanishing and enhance the robustness and convergence speed of the network. Finally, the fused features will be reorganized by a 1 × 1 convolution for channel reorganization and feature compression to achieve efficient integration of information. The structural design and functions of the three branches are described in the following sections.

**Large branch:** In this branch, a large kernel depth-separable convolution with a kernel size of K×K is employed to enhance the receptive field of features to more fully capture wide-range contextual information. Meanwhile, in order to further enrich the direction-awareness capability and mitigate the information loss problem, K×K convolutions parallel to 1×K and K×1 depth-separable convolutions are introduced for spatially dependent direction-decoupled modeling. Given that large kernel convolution will significantly increase the computational burden while enhancing the feature modeling capability, in this paper, the kernel size is set to K = 31 under the premise of ensuring the balance between the expression capability and efficiency, which effectively balances the receptive field expansion and computational resource constraints.

**Global branch:** Relying only on local convolution and multi-scale large kernel operation in the spatial domain, although it can take into account the regional features of small and large targets, it is difficult to take into account the global frequency information and long-distance dependency in the same model. To alleviate the above problems, this paper designs a global-awareness branch aiming to strengthen the model’s ability of modeling both the long-distance dependency and the global structure. In this branch, a dual-domain channel attention module (DCAM) is introduced to realize the complementary recalibration of features between the spatial and frequency domains to enhance the discriminative properties of the channel dimensions; at the same time, a frequency-domain guided multi-scale spatial attention module (FGM) is constructed to guide the generation of the multi-scale spatial attention through the introduction of the frequency-domain response information, which effectively improves the model’s capability of perception of the tiny targets in the complicated backgrounds and robustness, thus further optimizing the detection performance to adaptively modulate the global frequency distribution to realize the synergy between low-frequency structure and high-frequency details. The DCAM and FGMs are described in detail below. For a given input feature, $X_{G}\in R^{C\times H\times W}$, DCAM first applies the FCA module to the branch inputs of $X_{G}$, that is, DCAM first performs Fourier transform on $X_{G}$, and generates frequency-domain channel attention $W_{\mathrm{FCA}}$ through global average pooling GAP and 1×1 convolution, as shown below:

(13) $$X_{FCA}=F^{-1}\left(W_{FCA}⊗F\left(X_{G}\right)\right)$$

Among them, F represents Fast Fourier Transform (FFT), $F^{-1}$ represents Inverse Fast Fourier Transform (IFFT). After global modulation in the frequency domain, the obtained features will be sent to the Space Channel Joint Attention Module (SCA) to further explore their spatial distribution characteristics and inter channel dependencies, thereby enhancing the discriminative and effective feature expression.

(14) $$W_{\mathrm{SCA}}={\mathrm{Conv}}_{1\times 1}\left(\mathrm{GAP}\left(X_{\mathrm{FCA}}\right)\right)$$

The final output of the DCAM is obtained as:

(15) $$X_{\mathrm{DCAM}}=W_{\mathrm{SCA}}⊗X_{\mathrm{FCA}}$$

Then, through the FGM submodule, the shortcomings of spatial convolution in long-range dependencies and global pattern awareness are compensated for. The FGM submodule divides $X_{\mathrm{DCAM}}$ into two paths, $x_{1}=\mathrm{Dwconv}1\left(X_{DCAM}\right)$ is used for preserving spatial construction, $x_{2}=\mathrm{Dwconv}2\left(X_{DCAM}\right)$ calculates $F=F\left(x_{2}\right)$ in the frequency domain, and then obtains a frequency-domain-enhanced representation through the following operations:

(16) $$Z=\left|F^{-1}\left(x_{1}⊗\;F\right)\right|$$

Among them, |.| is an absolute value operation, ensuring that the features after inverse transformation are real numbers. Finally, the final output of the global branch is obtained by weighted fusion of residual and frequency-domain information using learnable parameters (α, β):

(17) $$Y_{g}=\alpha ⊗Z+\beta ⊗X_{DCAM}$$

Up to this point, the model can effectively focus on the frequency components with higher information density to achieve deep modeling and expression enhancement of image spectral features, thus improving the perception and discrimination performance of key visual cues such as texture details and structural changes.

**Local branch:** Finally, a 1×1 depthwise separable convolution is introduced for fine-grained modulation of local signals, as shown in Figure 6b.

### 3.5. SIoU Loss Function

The traditional CIoU [44] loss function lacks discriminative expression in scale modeling and does not model the directional differences between bounding boxes, which can easily cause gradient convergence oscillation and localization ambiguity during training. In order to further improve the resolution of spatial geometric modeling, this paper introduces the SCYLLA-IoU (SIoU) loss function [45]. SIoU models the relationship between predicted boxes and ground-truth boxes in four complementary dimensions. This direction-oriented geometric collaborative optimization mechanism not only enhances the sensitivity of the model to spatial structures, but also effectively alleviates the performance bottleneck of traditional loss functions in deformation target localization, ultimately achieving a more efficient and stable boundary box regression process.

**Figure 7:**
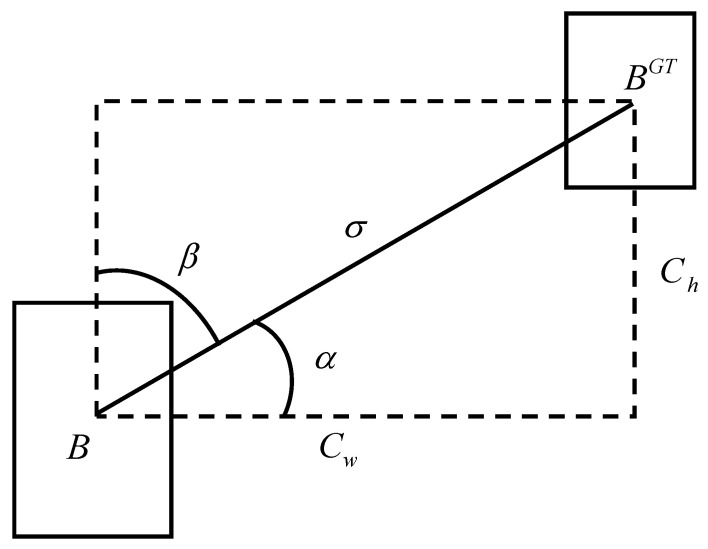
Angle loss calculation scheme.

Figure 7 shows the angular loss computational system, *B* is the prediction frame, $B^{GT}$ is the real frame, the horizontal distance between them is σ, and the vertical height difference is $C_{h}$. When the angle formed by the connecting vector between the centers of the predicted and ground-truth boxes falls below a specified threshold in the angular domain, the model tends to adjust the prediction frame along the direction of α, otherwise, it optimizes the prediction frame along the direction of β. The angle cost explicitly measures the difference in the angle between the line connecting the centers of the prediction frames and the horizontal (or vertical) axis, guiding the regression process to approach the target along the shortest axis first. The distance cost is based on the centroid offset and is designed in conjunction with the angular weights so that a larger offset angle corresponds to a larger penalty, minimizing the number of distance-related variables to make the prediction frame more accurate. The angular loss is calculated using the following formula.

(18) $$\Lambda =1-2\sin^{2}\left(\arcsin x-\frac{\pi }{4}\right)$$

The distance loss term measures the degradation in localization accuracy by modeling the centroid offset in 2D space. It introduces an angular weighting mechanism so that larger angular deviations correspond to larger penalties. When the direction of the offset is a large deviation from the centerline, the value of the loss is correspondingly larger, thus imposing a stronger cost on the large angular deviation to enhance the regression accuracy.

(19) $$\Delta =\sum_{t=x,y}\left(1-e^{-\rho \gamma t}\right)$$

Among them, *t* is the iteration variable, representing the *x*-axis and *y*-axis directions. γ is the adaptive weight coefficient for angle loss, and ρ is the normalized penalty term for center point deviation.

The shape loss is used to measure how much the predicted box deviates from the real box in terms of aspect ratio, and is intended to guide the model to adjust the geometry of the predicted box as quickly as possible to fit the proportional characteristics of the real target. This loss term drives the bounding box shape to align more accurately to the target structure by quantifying the degree of width and height match between the two. Its specific calculation is shown below:

(20) $$\Omega =\sum_{t=w,h}{\left(1-e^{-w^{t}}\right)}^{\theta }$$

Among them, θ represents the hyperparameter that controls the attention to shape loss.

The IoU loss term, on the other hand, serves as the core measure of spatial overlap quality, continues the traditional IoU calculation paradigm in its essence, and is used to measure the degree of regional consistency between the predicted frame and the real frame in the 2D plane. The loss reflects the geometric coverage relationship through the measure of the intersection ratio of the two, which has good scale invariance and target shape adaptation, and its formula is as follows:

(21) $$L_{\mathrm{IoU}}=1-\mathrm{IoU}$$

Ultimately, the SIoU (SCYLLA-IoU) loss function jointly constrains the positioning process of the bounding box from multiple dimensions by fusing the four geometric factors of angle, distance, shape and IoU into a unified optimization objective, which is formulated as follows:

(22) $$L_{\mathrm{SIoU}}=1-\mathrm{IoU}+\frac{\Delta +\Omega }{2}$$

When performing PCB defect detection tasks, the multidimensional geometric constraints of SIoU help the model converge faster, while enhancing the perception ability of small defect targets, improving overall detection accuracy and robustness.

## 4. Experiment and Analysis

### 4.1. Experimental Configuration

In terms of hardware configuration, the experiment used an Intel Core i5-12400F processor (Intel Corporation, Santa Clara, CA, USA) and NVIDIA RTX 4070 graphics card (ASUSTeK Computer Inc., Taipei, Taiwan) to build a testing platform, combined with PyTorch 2.0.1 deep learning framework. Among them, the image acquisition device for the DeepPCB dataset is a Linear Scan CCD Camera (resolution: 48 pixels per millimeter). The specific training parameter configuration is detailed in Table 1.

### 4.2. Dataset Preparation

1. PKU-Market-PCB dataset

**Figure 8:**
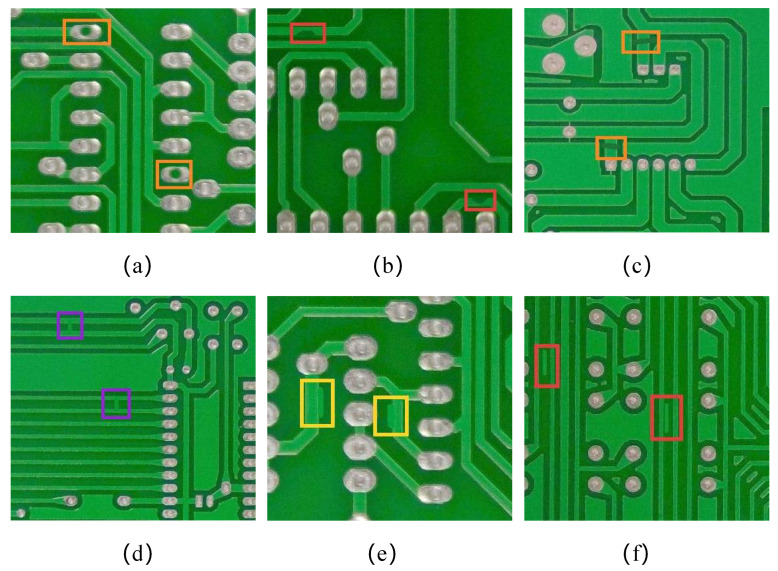
Six types of PCB defects: (**a**) Missing_hole; (**b**) Mouse_bite; (**c**) Open_circuit; (**d**) Short; (**e**) Spur; (**f**) Spurious_copper.

This paper uses the PKU-Market-PCB dataset publicly released by the Intelligent Robot Laboratory of Peking University, which is a public synthetic PCB dataset [46]. This dataset contains a total of 1386 PCB defect images, with an average resolution of 2777×2138 pixels. The types of defects are missing holes, mouse bites, open circuits, short circuits, burrs, and fake copper, as shown in Figure 8.

**Figure 9:**
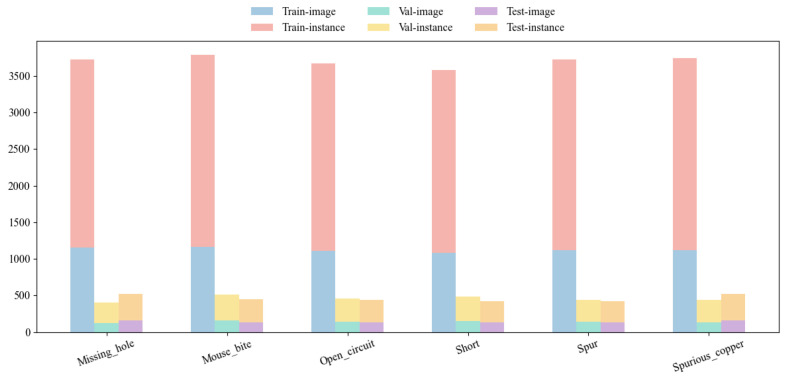
Category distribution of PKU-Market-PCB dataset.

**Figure 10:**
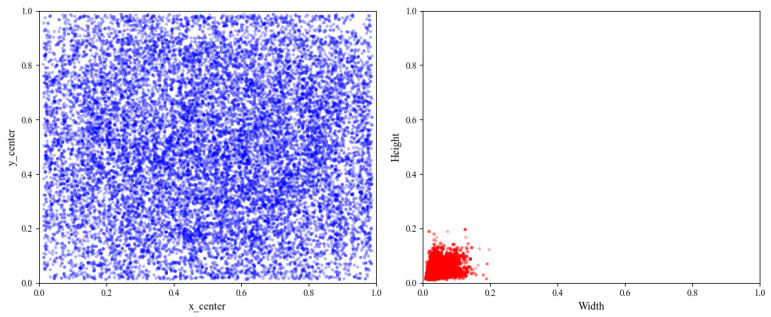
PKU-Market-PCB dataset target distribution map.

In order to improve the generalization ability of the model, this paper uses data augmentation techniques (Albumentations library) to randomly flip, rotate, crop, and cut the dataset, increasing the original sample size from 1386 to 8436, ensuring a relatively balanced distribution of each category. The dataset is strictly divided into training set, validation set, and testing set in an 8:1:1 ratio to ensure the objectivity of the evaluation results. Figure 9 shows the specific category distribution. Figure 10 shows the distribution of object sizes in the dataset, indicating that the dataset has the characteristics of small defect targets and a large number of defects.

2. DeepPCB dataset

**Figure 11:**
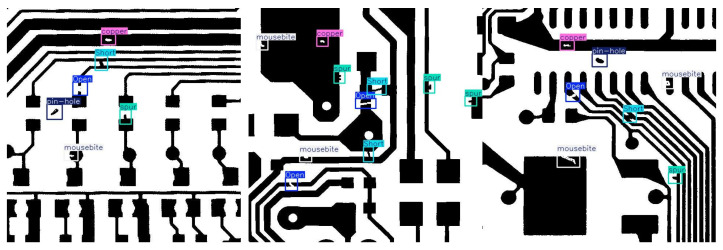
Six defect examples in DeepPCB dataset.

This paper also selected the DeepPCB dataset to further validate the generalization and robustness of our network [47]. All images in this dataset were obtained through a linear scanning CCD with a resolution of approximately 48 pixels per millimeter. The original image size is approximately 16k × 16k pixels, which is then cropped into sub images of 640 × 640, aligned using template matching techniques, and preprocessed using binarization and template matching techniques to reduce lighting interference. The DeepPCB dataset includes 1500 defective images and 1500 defect-free images. The dataset covers six typical PCB defects, including open circuit, short circuit, mouse bite, burr, copper dot, and hole, as shown in Figure 11. In all comparative experiments, we divided the dataset into a 7:2:1 ratio based on the training set, testing set, and validation set.

### 4.3. Evaluation Indicators

This paper uses strict COCO indicators to evaluate the detection performance of the model [48], including Precision (P), Recall (R), Mean Average Precision (mAP50), Mean Average Precision (mAP50-95), and F1 score. Here, mAP50 denotes the Mean Average Precision at an IoU threshold of 0.5, while mAP50-95 denotes the arithmetic mean of average Precision (AP) across 10 discrete gradients where the IoU threshold increases incrementally from 0.5 to 0.95 (with a step size of 0.05). Additionally, Params and GFLOPs are employed to evaluate the model’s parameter count and computational complexity. The mathematical formulas for each metric are as follows:

(23) $$P=\frac{TP}{TP+FP}$$

(24) $$R=\frac{TP}{TP+FN}$$

(25) $$AP=\int_{0}^{1}P\left(R\right)dR$$

(26) $$AP_{50-95}=\frac{AP_{50}+AP_{55}+\cdots +AP_{95}}{10}$$

(27) $$mAP_{50}=\frac{\sum_{i=1}^{n}AP_{50_{i}}}{n}$$

(28) $$mAP_{50-95}=\frac{\sum_{i=1}^{n}{\left[AP_{50-95}\right]}_{i}}{n}$$

(29) $$F1=\frac{2\times P\times R}{P+R}$$

Among these, TP (True Positive) represents the number of positive samples accurately identified by the model; FP (False Positive) denotes negative samples misclassified as positive; FN (False Negative) reflects the number of actual positive samples that were not detected. This paper introduces the F1 score, defined as the harmonic mean of Precision (P) and Recall (R), providing a comprehensive measure of the trade-off between Precision and Recall.

### 4.4. Experimental Results and Analysis

As shown in Table 2 and Table 3, the proposed model exhibits excellent performance on the PKU-Market-PCB and DeepPCB datasets, with high robustness and precise positioning ability.

### 4.5. Comparative Experiments

1. Comparative experimental results on the PKU-Market-PCB dataset

In order to systematically quantify the practical efficacy and relative advantages of the proposed model in the PCB defect detection task, this paper constructs comparison experiments with a variety of mainstream single-stage target detection frameworks, aiming to comprehensively and objectively evaluate their performance in terms of the dimensions of accuracy, robustness, and generalization ability, including Faster-RCNN [12], DINO [49], TOOD [50], YOLOv5 [51], YOLOv8 [52], YOLOv10 [53], YOLOv11 [52], GCC-YOLO [54], PD-YOLO [55] and Hsd-YOLO [56]. The comparison experiments use identical experimental conditions, including hardware and training parameters. Several evaluation metrics are used to evaluate all the comparison algorithms, including $AP_{50}$, AP, Params, GFLOPs, and FPS. The comparison results are shown in Table 4.

From the experimental results, the model proposed in this paper demonstrates excellent performance on the PKU-Market-PCB dataset. This method achieved 97.5% mAP50 and 59.5% mAP50-95, which are the highest among all comparative experiments, indicating that EP-YOLO has more outstanding detection ability for small defects in complex backgrounds. The detection performance of Faster RCNN and DINO two-stage algorithms on this dataset is far inferior to single-stage algorithms, and the computational cost is significantly high. In single-stage methods, the YOLO series models have significant advantages in speed, such as YOLOv11n achieving a high FPS (303) at a lower computational cost (6.3 GFLOPs), but its accuracy performance is relatively limited. The method proposed in this paper maintains high detection accuracy while controlling the computational complexity to 16.3 GFLOPs and achieving an inference speed of 158.7 FPS, achieving an ideal balance between accuracy and efficiency. Compared to YOLOv8s and YOLOv11s, our model not only achieves performance advantages, but also has significantly lower computational costs.

**Figure 12:**
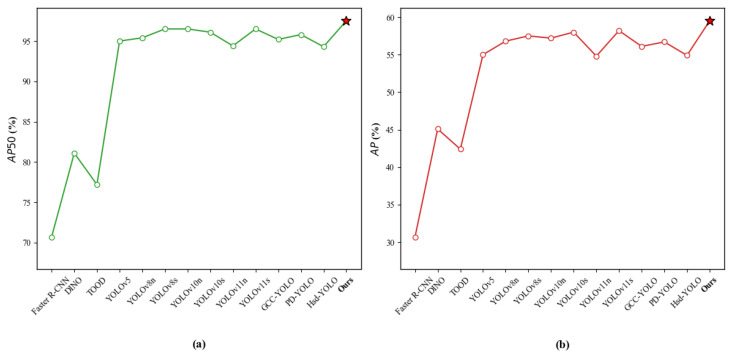
Comparison results of different algorithms in the PKU-Market-PCB dataset: (**a**) AP50; (**b**) AP.

On the PKU-Market-PCB dataset, Figure 12 provides a more intuitive performance comparison chart, which fully demonstrates the strong adaptability and detection capability of this method in PCB defect detection tasks.

2. Comparative experimental results on the DeepPCB dataset

**Figure 13:**
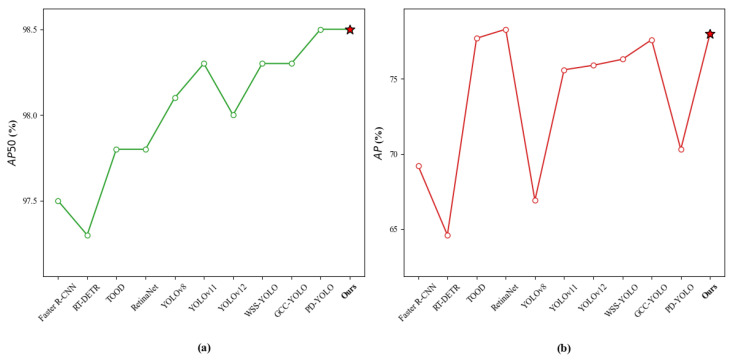
Comparison results of different algorithms in the DeepPCB dataset: (**a**) AP50; (**b**) AP.

To verify the generalization of the model, this paper conducted comparative experiments on the DeepPCB dataset, including Faster-RCNN [12], RT-DETR [37], TOOD [50], RetinaNet [57], YOLOv8 [52], YOLOv11 [52], YOLOv12 [58], WSS-YOLO [59], GCC-YOLO [54] and PD-YOLO [55]. The experimental results are shown in Table 5. The model in this paper achieved 78% on mAP50-95 and 98.5% on mAP50, ranking first among all compared models, proving the accuracy of the algorithm in target localization and recognition tasks. Although traditional two-stage methods such as Faster R-CNN achieve 97.5% on mAP50, their mAP50-95 is only 69.2%, and the computational complexity is as high as 816.13 GFLOPs, with an inference speed of only 3.89 FPS, which is difficult to meet real-time detection requirements. RT-DETR shows relatively low mAP values while improving inference efficiency (132.25 FPS). In the YOLO series methods, although some models perform better in terms of computational complexity and inference speed, the overall accuracy is still lower than the method proposed in this paper. Based on comprehensive analysis, the proposed model balances computational complexity (16.3 GFLOPs) and inference speed (136.05 FPS) while ensuring high detection accuracy, achieving a better balance between accuracy and efficiency. Figure 13 shows the comparison of algorithms under the mAP50 and mAP50-95 core metrics.

### 4.6. Ablation Experiments

In order to systematically verify the contribution of the proposed model architecture and its key components to overall performance, this paper conducted ablation experiments on the PKU-Market-PCB dataset to gradually analyze the actual effectiveness of various structural improvements in PCB defect detection tasks. The experimental results are shown in Table 6. In the single module experiment, CSPFOK brought the most significant performance improvement, with mAP50-95 increasing to 57.8% and F1 increasing to 95.0%, indicating its strong ability in feature expression. SCFE achieved an improvement in Precision (97.3%), indicating that it can effectively enhance feature discrimination ability. CCA has a relatively small impact on model complexity, and although the performance gain is relatively limited, the lack of CCA components will lead to a decrease in model performance.

In terms of multi module combination, the reconstructed detection head (+P2/−P5) and CSPFOK increased mAP50 to 96.5%, indicating that the combination of the two can improve feature utilization efficiency. On this basis, the introduction of SCFE further increased mAP50 to 96.8%, and mAP50-95 also reached the highest value among all configurations, indicating good complementarity between SCFE and CSPFOK. After further introducing CCA, the model Recall rate reached 94.8%, and mAP50 increased to 97.1%, but mAP50-95 slightly decreased, indicating that CCA is more inclined to improve Recall performance.

In Leave-one-out analysis, each component contributes to the performance of the model to varying degrees, and regardless of which component is removed, it has a certain impact on the performance of the model. Among them, the improvement of mAP value by the reconstructed detection head (+P2/−P5) is the most critical. Once removed, it will lead to a significant decrease in performance. This is mainly due to the introduction of the P2 detection layer, which can effectively preserve high-resolution feature information and enhance the model’s perception ability of small target defects. CCA is mainly used to improve Recall rate and has a relatively limited impact on overall accuracy. Overall, the modules have good complementarity, and the final model achieved more balanced and stable performance in multiple indicators, thus verifying the effectiveness and rationality of the proposed method.

**Figure 14:**
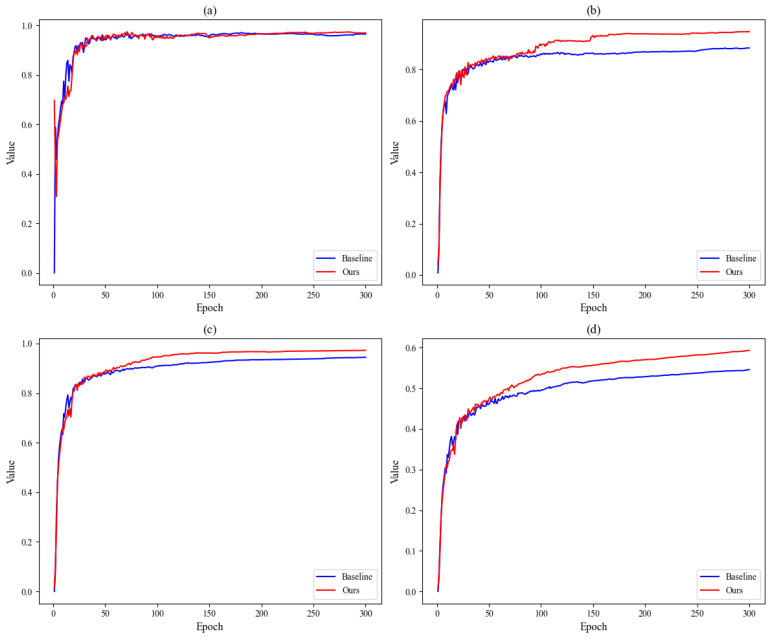
Performance trend curves during model training: (**a**) Precision; (**b**) Recall; (**c**) mAP50; (**d**) mAP50-95.

Figure 14 shows the trend of key indicators during the model training process before and after improvement. It can be seen that by the time the model reaches 300 epochs of training, mAP50 has already converged, and the curves of each metric remain at a high level. This indicates that the network in this paper has achieved better training results.

## 5. Discussion

### 5.1. Analysis of the Effectiveness of Refactoring the Detection Head

The reconstructed detection head significantly improves the model’s perception ability of small defect targets by introducing low-level P2 detection heads and removing high-level P5 branches. During multiple downsampling processes in YOLOv8, the detection heads corresponding to larger receptive fields (such as P5) are prone to severe loss of semantic and positional information, which has a negative impact on fine-grained localization tasks. Moving the detection center down to the shallow feature layer helps to better preserve key texture and edge information in the image. This paper refers to the method in reference [20] and combines the P2, P3, P4, and P5 detection head systems for multiple comparative experiments. The results are shown in Table 7. It can be seen that adding P2 detection head and removing P5 detection head has the best detection effect.

### 5.2. Effectiveness Analysis of CSPFOK

The design of a CSPFOK module is mainly used to achieve more efficient feature expression. With the appropriate increase in GFLOPs and Params, CSPFOK increases accuracy by 0.4%, Recall by 1.4%, mAP50 by 0.7%, and mAP50-95 by 1.7%, effectively balancing computational efficiency and detection accuracy.

In order to rigorously demonstrate the effectiveness of CSPFOK and the rationality of its hyperparameter selection, this paper conducted ablation experiments on the hyperparameters *K* and *e* of the CSPFOK module, and the experimental results are shown in Table 8 and Table 9. In the selection of large nuclear size *K*, as *K* increased from 7 to 31, mAP50 increased from 95.7% to 96.4%, effectively verifying the critical role of large receptive fields in capturing industrial defect features. As *K* continues to increase to 39, although the accuracy slightly improves, the computational cost also further increases. Therefore, *K* = 31 achieves a good balance between detection performance, computational cost, and efficiency. Meanwhile, the analysis of the channel ratio e for CSP segmentation shows that when *e* = 0.25, the model achieved higher mAP50 (96.4%) and mAP50-95 (57.8%) at a reasonable computational cost (19.9 GFLOPs). Compared to the full channel scheme with *e* = 1, the model not only had fewer parameters and computational costs, but also increased FPS by about 25.1%, avoiding overfitting risks caused by redundant calculations. The experimental results also demonstrate that the full channel scheme with *e* = 1 leads to a decrease in detection performance. The experimental results demonstrate that the hyperparameter selection with *K* = 31 and *e* = 0.25 is correct.

**Figure 15:**
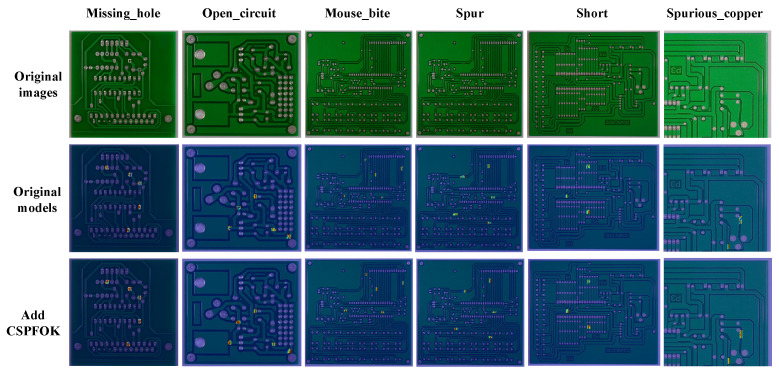
Comparison of heat maps before and after adding CSPFOK module.

This paper presents the visualization results of the heatmap before and after replacing the CSPFOK module, as shown in Figure 15. It can be seen that the model after replacement pays more attention to the defect area and shines brighter, especially the difference in impurity copper is more obvious. These conclusions demonstrate that the CSPFOK module achieves collaborative modeling of local spatial information and global semantic information, effectively enhancing feature extraction performance.

### 5.3. Effectiveness Analysis of SCFE

The core of the SCFE module is to downsample the shallow feature maps P2 and P3 through SPDConv, thereby improving the multi-scale feature fusion strategy. This paper designed an ablation experiment: based on the reconstruction of the detection head architecture (+P2/−P5), SPDConv was replaced with traditional convolution operation for downsampling, and its performance was evaluated. The results are shown in Table 10. Experiments have shown that the use of SPDConv significantly improves the detection of defects such as “Mouse_ bite”, “Spur”, and “Spurious_ copper”. This result confirms the crucial role of SPDConv in enhancing feature expression ability during the downsampling process.

### 5.4. Effectiveness Analysis of CCA

In order to further evaluate the actual contribution of CCA modules in PCB defect detection, based on the reconstruction of the detection head architecture (+P2/−P5), the CoordAtt attention mechanism was further replaced with several typical attention structures, including EMA [60], SimAM [61], and CBAM [62], and comparative experiments were conducted. The specific results are shown in Table 11.

The experimental results indicate that although EMA and SimAM have slightly better accuracy metrics than CoordAtt, they have certain disadvantages in terms of Recall rate. Specifically, CoordAtt’s Recall rate reached 96.5%, significantly higher than other attention mechanisms, and its F1 score was also the highest. This result fully demonstrates that CoordAtt has more advantages in the joint modeling of channel information and spatial position information, which can effectively alleviate missed detections while maintaining detection accuracy, thereby achieving more robust and discriminative feature expression.

**Figure 16:**
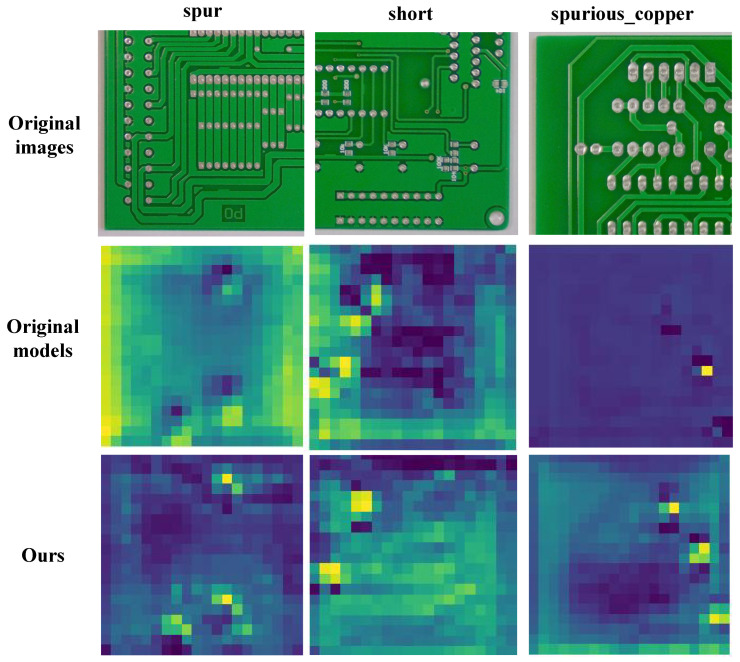
Comparison of feature mapping before and after CCA addition.

Figure 16 shows the feature mapping before and after adding the CCA module. It can be clearly felt that after adding CCA, the highlighted areas in the feature map are more focused on the true position of the defect, which effectively proves that the addition of CCA enhances the detection performance of the model for small defects.

### 5.5. Heat Map Analysis on DeeppCB Dataset

**Figure 17:**
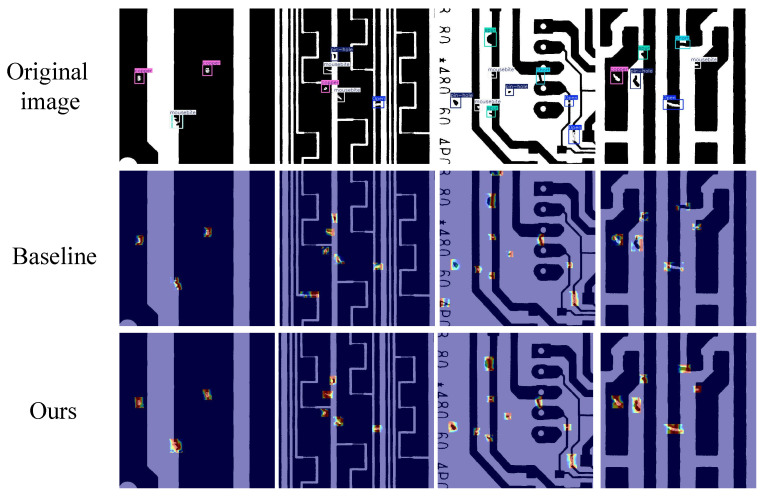
Heat map comparison.

In order to more intuitively demonstrate the advantages of our model compared to the baseline model, we provide a heatmap comparison on the DeepPCB dataset, as shown in Figure 17. The brighter and denser it is, the stronger the model’s focus on defect location. It can be seen that the brightness of the real defect location in this paper’s model is brighter and more concentrated, while the brightness of the baseline model is relatively dark and divergent. Moreover, the baseline model detected false positives in the images of the second and third columns, which means that it also performed heatmap detection on positions without defects. This paper’s model can correctly detect defect targets, proving that the improvement of this paper’s model is effective.

### 5.6. Analysis of the Impact of Different Loss Functions

This paper conducted comparative experiments on the impact of different loss functions on model performance in the PKU-Market-PCB dataset, and the experimental results are shown in Table 12. The SIoU loss function mAP50 used in this paper reaches 97.5%, which is superior to CIoU (97.1%), DIoU (97.1%), EIoU (96.6%), and WIoU (96.9%), and SIoU has the highest mAP50-95 (59.5%). This is mainly attributed to the introduction of angle cost between the predicted box and the real box by SIoU. In the PCB defect detection scenario, SIoU effectively reduces the degrees of freedom in the regression process and improves the accuracy of localization by considering vector angles for small and irregularly distributed defects. Although the FPS of SIoU (158.7) is slightly lower than that of CIoU (165.5), it is worthwhile for the overall performance improvement of the model. The experimental results indicate that selecting SIoU can make EP-YOLO more robust in dealing with small targets in complex backgrounds.

### 5.7. Discussion of Model Inference

After the model training converges, this paper deploys the optimized EP-YOLO network to perform inference operations on the test set to evaluate its generalization performance in real defect detection tasks, and the inference parameters are shown in Table 13; conf is the confidence threshold below which the detection results are filtered, controlling whether the low-confidence targets are retained or not; NMS IoU controls whether the overlapping frames are kept or not. Although most of the images were able to successfully detect the target defects, there were still cases where some images failed to detect any defects. After analyzing, the possible reasons include the following two main aspects:

(1) The first may be the limited number of defect type samples, which leads to the model’s insufficient feature learning for these categories and limited generalization ability. The model may not be able to accurately recognize the more rare or fuzzy boundary defects when they appear in the test image. Second, because PCB defects often have small scale, irregular shape, and insignificant color contrast, resulting in some of the defects not being obvious in the image and easily ignored by the model.

(2) The confidence threshold for model inference was set to 0.25 and the NMS IoU threshold was set to 0.7. By adjusting these two parameters in the experiment, it was found that no matter how small or large the confidence threshold and the IOU threshold were set to, those pictures with undetected defects were always not detected with defects, and this result ruled out the possibility of leakage due to the improper threshold settings (conf, NMS IoU). Therefore it is possible that the model itself is limited, although EP-YOLO improves the small target detection ability to a certain extent, it still has difficulty in recognizing some defects with low contrast and fuzzy boundaries. This indicates that there are still limitations in the feature extraction capability of the current model.

## 6. Conclusions and Prospects

This study focuses on the defect detection task of printed circuit boards (PCBs) and proposes an improved model with excellent performance, EP-YOLO, based on the YOLOv8 detection framework. This method effectively reduces the number of network parameters while improving detection accuracy. Specifically, this paper first reconstructs the detection head to enhance its responsiveness to small targets; at the same time, introducing the SCFE network to enhance shallow semantic representation and further improve the information exchange mechanism of multi-scale features. Secondly, this paper designed CCA and CSPFOK modules, effectively addressing challenges such as small target size and complex background in PCB images, and improving the effectiveness of feature extraction and expression. Finally, Using SIoU, the bounding box regression training is improved by integrating angle optimization and dynamic focusing mechanism, thereby enhancing convergence speed and detection performance. The experimental results showed that EP-YOLO achieved 97.5% mAP50 on the PKU-Market-PCB dataset and 98.5% mAP50 on the DeepPCB dataset, with a parameter reduction of approximately 12.55%. The frame rate (FPS) reaches 158.7, meeting the dual requirements of accuracy and speed in actual industrial testing scenarios.

Although EP-YOLO has made significant progress in performance, it still faces certain challenges in actual industrial deployment, especially in scenarios with limited computing resources or edge devices. Therefore, further reducing model complexity and operational load while ensuring detection accuracy is a key direction for future optimization. In addition, to enhance the cross-platform adaptation capability of the model, customized optimization should be carried out in conjunction with the target hardware architecture to ensure the stability and robustness of the model in diverse industrial environments. On the other hand, the model still has the problem of missing extremely small targets or weak texture defects during the inference stage. To address this issue, follow-up work can start from the data dimension, by expanding the training set size, introducing synthetic defect generation, adopting self-supervised learning and more complex data augmentation strategies to enhance the model’s generalization ability, thereby enhancing the practicality and engineering value of the model.

## Figures and Tables

**Table 1:** Configuration parameters used in our experiments.

Parameter	Configuration
GPUs	NVIDIA GeForce RTX 4070
Operating System	Windows 10
PyTorch	2.0.1
CUDA	11.8
Epochs	300
Batch Size	16
Input Size	640 × 640
Optimizer	SGD
Learning Rate Weight	0.01
Decay Momentum	0.0005
Momentum	0.937
Image acquisition device for DeepPCB dataset	Linear Scan CCD Camera
Data augmentation library	Albumentations

**Table 2:** Test results for defect detection in PKU-Market-PCB dataset.

Defect Type	Missing_Hole	Mouse_Bite	Open_Circuit	Short	Spur	Spurious_Copper
Precision (%)	95.3	96.9	96.3	96.9	97.5	94.5
Recall (%)	97.3	95.2	95.0	98.7	91.1	92.7
$AP_{50}$ (%)	98.3	97.1	98.6	99.2	95.7	95.9
AP (%)	63.2	58.7	59.0	60.3	58.2	57.4

**Table 3:** Test results for defect detection in DeepPCB dataset.

Defect Type	Open	Short	Mousebite	Spur	Pin-Hole	Copper
Precision (%)	96.4	96.7	97.9	98.5	97.0	94.2
Recall (%)	97.0	94.4	96.0	94.9	98.7	97.4
$AP_{50}$ (%)	98.3	98.3	98.3	98.7	99.2	98.5
AP (%)	71.2	68.2	76.5	76.6	88.8	87.0

**Table 4:** Comparative experimental results of different algorithms in PKU-Market-PCB dataset.

Method	${\mathrm{AP}}_{50}$ (%)	AP (%)	GFLOPs	Params (M)	FPS
Faster R-CNN	70.7	30.7	816.1	33.08	3.90
DINO	81.1	45.1	220.2	47.55	11.79
TOOD	77.2	42.4	155.7	32.03	48.74
YOLOv5	95.0	55.0	7.1	2.50	294.00
YOLOv8n	95.4	56.8	8.2	3.01	312.50
YOLOv8s	96.5	57.5	28.4	11.13	182.28
YOLOv10n	96.5	57.2	6.5	2.27	357.00
YOLOv10s	96.1	58.0	21.4	7.22	209.33
YOLOv11n	94.4	54.8	6.3	2.58	303.00
YOLOv11s	96.5	58.2	21.3	9.42	193.50
GCC-YOLO	95.2	56.1	11.0	2.38	195.24
PD-YOLO	95.8	56.7	10.8	2.63	206.33
Hsd-YOLO	94.3	54.9	7.6	2.61	196.48
Ours	97.5	59.5	16.3	2.63	158.70

**Table 5:** Comparative experimental results of different algorithms in DeepPCB dataset.

Method	${\mathrm{AP}}_{50}$ (%)	AP (%)	GFLOPs	Params (M)	FPS
Faster R-CNN	97.5	69.2	816.1	33.09	3.89
RT-DETR	97.3	64.6	57.0	19.90	132.25
TOOD	97.8	77.7	125.9	32.03	19.82
RetinaNet	97.8	78.3	97.5	19.88	50.91
YOLOv8n	98.1	66.9	8.1	3.01	228.80
YOLOv11n	98.3	75.6	6.3	2.58	168.86
YOLOv12n	98.0	75.9	6.3	2.56	176.85
WSS-YOLO	98.3	76.3	5.4	2.09	179.97
GCC-YOLO	98.3	77.6	11.0	2.38	176.48
PD-YOLO	98.5	70.3	10.8	2.63	160.31
Ours	98.5	78.0	16.3	2.63	136.05

**Table 6:** Ablation experiments on the PKU-Market-PCB dataset.

Model	+P2/−P5	CSPFOK	SCFE	CCA	SIoU	P (%)	R (%)	${\mathrm{AP}}_{50}$ (%)	AP (%)	GFLOPs	Params (M)	F1 (%)	FPS
Baseline						95.6	91.5	95.4	56.8	8.2	3.01	93.5	312.5
1	✓					95.9	91.5	95.8	56.9	11.7	2.03	93.6	227.0
2		✓				97.0	93.1	96.4	57.8	19.9	4.88	95.0	185.1
3			✓			97.3	92.0	95.7	57.0	10.0	3.31	94.6	256.2
4				✓		97.0	92.1	95.9	56.7	8.1	3.02	94.5	258.1
5	✓	✓				96.3	92.9	96.5	58.6	13.2	2.14	94.6	188.7
6	✓	✓	✓			97.3	93.0	96.8	60.7	16.3	2.62	95.1	169.5
7	✓	✓	✓	✓		96.9	94.8	97.1	59.4	16.3	2.63	95.8	165.5
8		✓	✓	✓	✓	97.0	91.4	96.0	57.6	12.2	3.58	94.2	207.4
9	✓		✓	✓	✓	96.3	94.5	96.8	58.1	13.7	2.43	95.4	200.2
10	✓	✓		✓	✓	96.7	93.8	97.1	59.0	12.8	2.12	95.2	180.3
11	✓	✓	✓		✓	96.4	93.5	96.7	58.8	15.8	2.56	94.9	186.7
12	✓	✓	✓	✓	✓	96.2	95.0	97.5	59.5	16.3	2.63	95.6	158.7

**Table 7:** Performance comparison of different detection head configurations.

Different Head Combinations	${\mathrm{AP}}_{50}$ for Different Types of Defects (%)						
	Missing_Hole	Mouse_Bite	Open_Circuit	Short	Spur	Spurious_Copper	All
P2 + P3 + P4	98.3	97.1	98.6	99.2	95.7	95.9	97.5
P3 + P4	98.8	96.2	96.3	98.6	94.2	94.4	96.4
P3 + P4 + P5	98.1	95.1	96.1	99.4	94.0	94.5	96.2
P2 + P3 + P4 + P5	98.7	97.0	98.7	99.2	94.3	94.9	97.2

**Table 8:** Ablation study on different K settings of CSPFOK.

K	P (%)	R (%)	${\mathrm{AP}}_{50}$ (%)	AP (%)	GFLOPs	FPS	Params (M)	F1 (%)
K = 7	97.0	90.7	95.7	57.9	19.4	212.39	4.83	93.8
K = 15	95.8	92.3	95.9	57.2	19.5	208.14	4.84	94.0
K = 31	97.0	93.1	96.4	57.8	19.9	185.07	4.88	95.0
K = 39	97.2	93.4	96.6	57.9	20.3	180.12	4.91	95.3

**Table 9:** Ablation study of channel scaling factor *e*.

*e*	P (%)	R (%)	${\mathrm{AP}}_{50}$ (%)	AP (%)	GFLOPs	FPS	Params (M)	F1 (%)
0.125	96.4	92.7	96.3	57.6	19.5	219.98	4.85	94.5
0.25	97.0	93.1	96.4	57.8	19.9	185.07	4.88	95.0
0.50	96.9	93.2	96.6	58.2	20.9	178.43	4.97	94.9
0.75	96.2	93.6	96.8	58.2	22.1	155.48	5.09	95.0
1.00	96.6	92.6	96.2	57.1	23.6	138.69	5.24	94.5

**Table 10:** Results of SCFE ablation experiment.

SPDConv	Missing Hole	Mouse Bite	Open Circuit	Short	Spur	Spurious Copper	All
	98.0	94.1	95.5	98.7	93.6	93.0	95.5
✓	97.5	95.9	95.4	99.0	94.5	95.5	96.3

**Table 11:** Comparative results of attention mechanisms.

Attention	Precision (%)	Recall (%)	${\mathrm{AP}}_{50}$ (%)	F1
+EMA	97.1	91.9	96.0	94.4
+CBAM	96.5	92.1	96.1	94.2
+SimAM	97.1	91.0	96.1	94.0
+CoordAtt	96.7	92.4	96.5	94.5

**Table 12:** Ablation study of different IoU loss functions on model performance.

IoU Type	P (%)	R (%)	${\mathrm{AP}}_{50}$ (%)	AP (%)	FPS
CIoU	96.9	94.8	97.1	59.4	165.50
DIoU	96.8	94.2	97.1	58.1	151.79
EIoU	95.7	93.6	96.6	58.0	132.41
WIoU	96.2	94.6	96.9	57.2	148.57
SIoU (Ours)	96.2	95.0	97.5	59.5	158.70

**Table 13:** Inference parameter configuration.

Parameter	Configuration
Data Source	Test Data
Input size	640 × 640
conf	0.25
NMS IoU	0.7

## Data Availability

The original contributions presented in the study are included in the article, further inquiries can be directed to the corresponding author.
